# Complete Genome Sequence of the Original *Escherichia coli* Isolate, Strain NCTC86

**DOI:** 10.1128/genomeA.00243-17

**Published:** 2017-04-20

**Authors:** Varnica Khetrapal, Kurosh S. Mehershahi, Swaine L. Chen

**Affiliations:** a National University of Singapore, Singapore; b Genome Institute of Singapore, Singapore

## Abstract

*Escherichia coli* is the most well-studied bacterium and a common colonizer of the lower mammalian gastrointestinal tract. We report here the complete genome sequence of the original *Escherichia coli* isolate, strain NCTC86, which was described by Theodor Escherich, for whom the genus is named.

## GENOME ANNOUNCEMENT

*Escherichia coli* is the most important and best-studied bacterial model system, having been used to derive seminal understanding of bacterial genetics, regulation, evolution, and virulence. *E. coli* is eponymously named for Theodor Escherich, a German physician who isolated the bacterium from the stool of infants (1, 2). Escherich’s isolate was originally referred to descriptively as *Bacillus coli commune*; the name *Escherichia coli* was proposed in 1919 (3) and officially accepted in 1958 (4). In 2007, the 150th anniversary of Escherich’s birth was honored, leading to reflections on his career and subsequent impact (5). More recently, a complete genome sequence performed by Illumina sequencing was published along with a concise history of the isolation and early history of the original *B. coli commune*, which eventually was stored in the United Kingdom National Collection of Type Cultures as NCTC86 (6). An initial genomic analysis indicated that NCTC86 is fairly typical of nonpathogenic commensal *E. coli* frequently found in humans. This initial genome assembly consisted of 314 contigs with an *N*_50_ size of 32,645 bp (6). We therefore used PacBio sequencing to derive a complete assembly and methylome of the NCTC86 genome.

NCTC86 genomic DNA was sheared to a size of 10 kb using a g-Tube (Covaris). A SMRTbell library was prepared according to the manufacturer’s instructions, loaded with a MagBead bound library protocol, and sequenced on two SMRTCells using P5-C3 chemistry on the PacBio RS II instrument (Pacific Biosciences) with a 180-min movie time. *De novo* assembly was performed with the Hierarchical Genome Assembly Process (HGAP3) in the SMRT Analysis version 2.3 suite using default parameters, except that the minimum seed read length was set to 7000 and the overlapper error rate was set to 0.04 (7). The sequence was polished using data from the same SMRTCells using the resequencing protocol. There were 80,396 reads and 857,099,100 nucleotides that passed filtering (approximate coverage of 140×), with a preassembly mean read length of 10,660 bp.

The NCTC86 genome consists of a single circular chromosome of 5,111,920 bp with a G+C content of 50.66%. The NCTC86 genome was annotated using the NCBI Prokaryotic Genome Annotation Pipeline (8). The NCTC86 chromosome contains 4,934 protein-coding sequences, 22 rRNA genes, and 87 tRNA genes. DNA methylation was analyzed with the modification and motif protocol in the SMRT Analysis version 2.3 suite using default parameters. An analysis by REBASE (9) indicated that NCTC86 contains the Dam and Dcm methylases, a type IIγ restriction methylase methylating GGT**A**CC motifs (6 mA at the bolded nucleotide), and a predicted type III restriction methylase methylating GAG**C**C motifs (4 mC at the bolded C). The finished genome sequence of *E. coli* strain NCTC86 will provide for historical context a high-quality reference sequence of the most important bacterial laboratory model system, as well as facilitate further genetic studies into basic bacterial regulation and disease mechanisms.

### Accession number(s).

The genome sequence of the *E. coli* strain NCTC86 chromosome has been submitted to GenBank (GenBank accession no. CP019778, SRA accession no. SRR5264437, BioProject no. PRJNA373816). Methylation data were submitted to REBASE (http://rebase.neb.com/cgi-bin/onumget?16605).
